# Favorable control of rapidly progressive retroperitoneal pleomorphic leiomyosarcoma with multimodality therapy: a case report

**DOI:** 10.1186/1756-0500-7-377

**Published:** 2014-06-19

**Authors:** Kosuke Sagara, Kotoe Takayoshi, Eiji Kusumoto, Keita Uchino, Taisei Matsumura, Hitoshi Kusaba, Seiya Momosaki, Koji Ikejiri, Eishi Baba

**Affiliations:** 1 Department of Medical Oncology, Clinical Research Institute, National Hospital Organization Kyushu Medical Center, 1-8-1 Jigyouhama, Chuo-ku, Fukuoka 810-8563, Japan; 2 Department of Gastrointestinal Surgery, National Hospital Organization Kyushu Medical Center, Fukuoka, Japan; 3 Department of Radiology, National Hospital Organization Kyushu Medical Center, Fukuoka, Japan; 4 Department of Pathological Department, National Hospital Organization Kyushu Medical Center, Fukuoka, Japan; 5 Department of Medicine and Biosystemic Science, Kyushu University Graduate School of Medical Sciences, Fukuoka, Japan; 6 Department of Comprehensive Clinical Oncology, Faculty of Medical Sciences, Kyushu University, Fukuoka, Japan

**Keywords:** Leiomyosarcoma, Local control, Multimodality therapy, Chemotherapy

## Abstract

**Background:**

Retroperitoneal sarcomas (RPS), such as pleomorphic leiomyosarcoma, often invade or displace vital organs in the abdominal cavity and exhibit an aggressive clinical course. Complete surgical resection of the tumor and preoperative radiotherapy and chemotherapies can be used for non-metastatic RPS. However, in case of huge retroperitoneal sarcoma fully occupying the abdominal cavity, surgical resection tends to be insufficient, resulting in poor outcomes. This report describes a case of rapidly progressive retroperitoneal pleomorphic leiomyosarcoma that was favorably controlled by debulking surgery followed by combination chemotherapy and radiotherapy.

**Case presentation:**

A 65-year-old Japanese woman developed abdominal discomfort due to a huge retroperitoneal tumor fully occupying the abdominal cavity. The immunohistochemical diagnosis was pleomorphic leiomyosarcoma with high-grade malignancy and aggressive proliferative features. Debulking surgery could be performed, but the small residual tumor had rapidly grown to an approximately 22 cm in length on the major axis within 38 days after the operation. The patient’s general condition progressively declined. Combination chemotherapy, consisting of doxorubicin and ifosfamide, was successfully administered for six cycles while maintaining dose intensity. The best objective response was a partial response, and the chemotherapy was well tolerated. Approximately 50 Gy of radiotherapy was delivered to the remaining tumor. This multimodal strategy resulted in progression-free survival for more than 17 months and achieved sustained symptomatic relief.

**Conclusions:**

Multimodal therapy with debulking surgery, combination chemotherapy and radiotherapy controlled a rapidly progressive retroperitoneal pleomorphic leiomyosarcoma. Maintaining dose intensity of the chemotherapy and radiotherapy might contribute to overall tumor control.

## Background

Soft tissue sarcomas (STS) are malignant tumors that arise from mesoderm-derived tissues, such as fat, muscle, and connective tissue. Approximately 15% of cases of STS occur in the retroperitoneum. Since retroperitoneal sarcomas (RPS) are often asymptomatic until they become large and are located adjacent to vital organs and structures (e.g., the aorta, inferior vena cava, duodenum, and head of the pancreas), appropriate surgical resection with negative microscopic margins (R0 resection) is sometimes difficult to achieve. Furthermore, even after curative resection is achieved, more than half of RPS patients develop local recurrence, which is the leading cause of death [1,2]. Multimodal therapy incorporating surgery, radiotherapy, and chemotherapy is thus expected to improve survival in patients with RPS. We treated an RPS patient with rapidly progressive features of pleomorphic leiomyosarcoma (PLS) using combined modalities and achieved relatively long-term disease control.

## Case presentation

**Figure 1:**
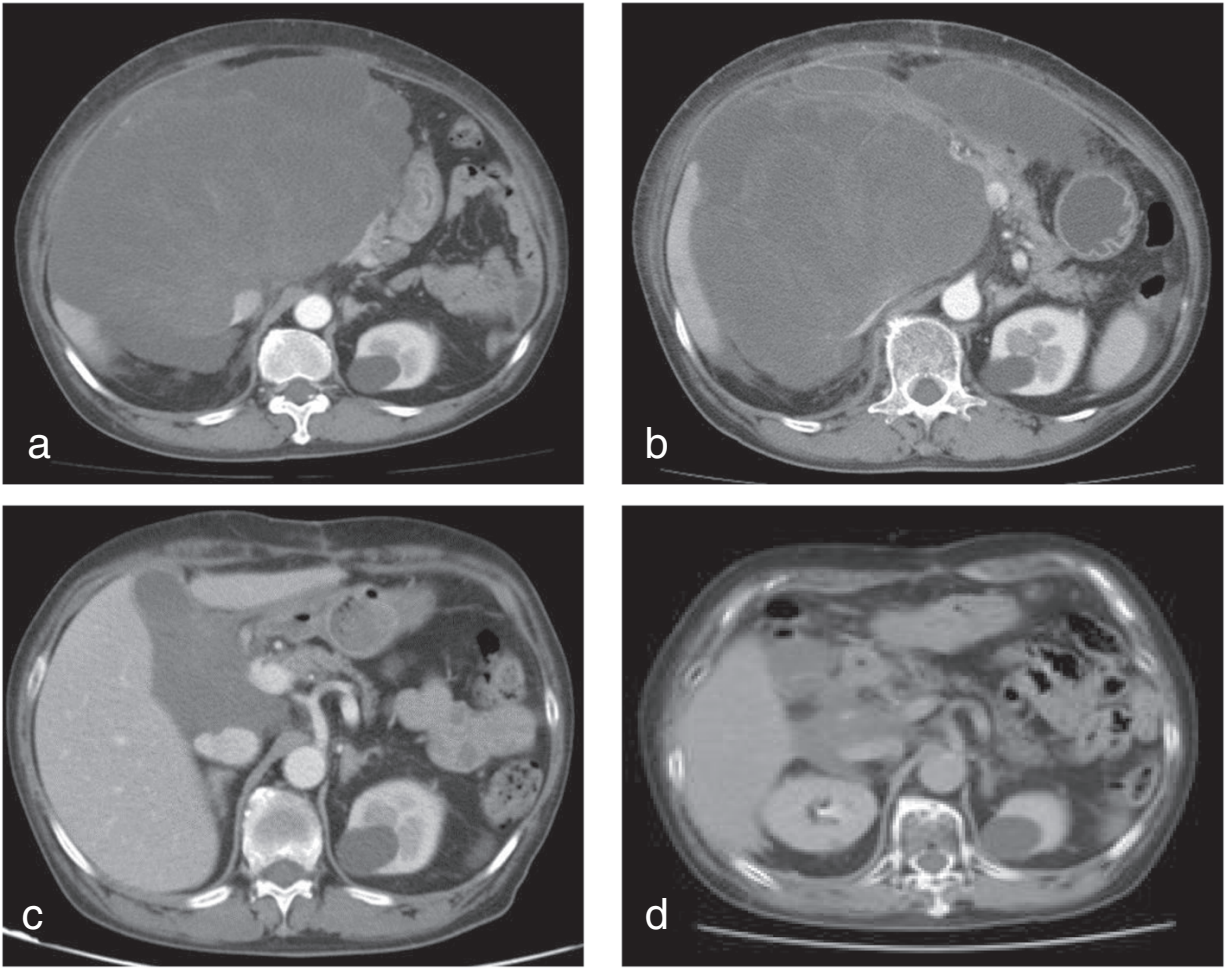
**Computed tomography scan of the abdominal tumor. (a)** Computed tomography (CT) scan at the time of the initial patient visit. A CT scan obtained before the operation reveals a bulky 30 × 23 × 15 cm mass in the right retroperitoneal space, ranging from the undersurface of the liver to the pelvis. The tumor contained a clustered contrast-filled bulkhead and cyst-like structure. **(b)** CT scan performed at 38 days after the debulking surgery. At 38 days after successfully performed debulking surgery, the formerly small residual tumor is noted to have progressed rapidly, returning to the size of the tumor observed before surgery. **(c)** CT scan after chemotherapy. CT scan shows reduction in tumor size in response to five cycles of chemotherapy with doxorubicin and ifosfamide. **(d)** CT scan after radiotherapy. The residual tumor continued to shrink following the completion of radiation therapy.

**Figure 2:**
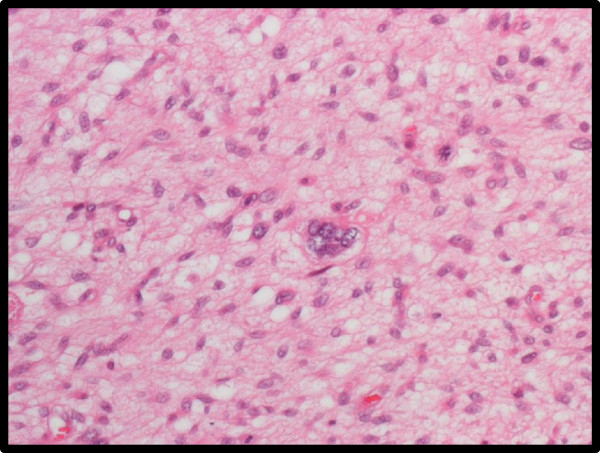
**Pathological examination of the resected tumor.** Pathological examination of the resected tumor shows multiple nuclei in the center and abnormal mitosis in the upper right area of this image. These findings indicate high-grade malignancy.

A 65-year-old Japanese female presented to our department with abdominal discomfort. Computed tomography (CT) revealed a 30 × 23 × 15 cm tumor in the right retroperitoneum, compressing the inferior vena cava (Figure 1a). The patient had no relevant personal or family history. Laboratory blood testing showed no major signs of organ dysfunction, except for increased levels of aspartate aminotransferase (AST), alanine aminotransferase (ALP), and γ-glutamyltranspeptidase (γ-GTP). Preoperative radiotherapy was not administered because a large treatment volume could otherwise lead to late radiation-related damage of critical organs, such as the kidneys, liver and small bowel and because the patient required a therapeutic strategy that would produce more rapid relief of symptoms. Surgical resection for the purpose of tumor debulking was then performed. A small tumor beside the anterior aspect of the inferior vena cava and on the uncinate process of the pancreas that was approximately 2 cm in length on the major axis remained. Immunohistochemical analysis revealed high-grade malignant cells strongly positive for h-caldesmon and positive for α-smooth muscle actin, desmin, and calponin, resulting in a diagnosis of PLS. A high proliferation rate of the tumor cells was also seen on immunohistochemical analysis (Figure 2).

During the period of her recovery from the surgery and preparation for adjuvant radiotherapy, abdominal fullness recurred, and a CT scan revealed that the tumor had regrown to 22 × 14 × 23 cm within only 38 days after the surgery. The regrown tumor was approximately 1000-fold higher in volume when compared with the residual tumor (Figure 1b). On day 47 after the operation, combination chemotherapy with doxorubicin (DXR) and ifosfamide (IFM) was initiated. The dose administration schedule was designed as follows: infusional DXR (30 mg/m^2^) on days 1–2 and IFM (2,000 mg/m^2^) on days 1–5 every 21 days. The chemotherapy was administered for six cycles, and her symptoms decreased while Eastern Cooperative Oncology Group performance status (PS) improved by the time of the initiation of the second cycle. The best objective response rate was a partial response (PR, 64%; 22 × 14 × 23 cm before chemotherapy and 8 × 8 × 13 cm after 5 cycles of chemotherapy) according to the new response evaluation criteria in solid tumours (RECIST) ver. 1.1 (Figure 1c). Common terminology criteria of adverse events (CTC-AE) ver. 4 grade 4 neutropenia and grade 3 febrile neutropenia occurred during the first cycle. In subsequent cycles of chemotherapy, the prophylactic administration of granulocyte-colony stimulating factor (filgrastim, 75 μg/day) was employed to maintain the dose intensity of the chemotherapy. The chemotherapy was generally well tolerated. After the fifth cycle, the tumor remained beside the inferior vena cava, and the patient received a conventional radiotherapy dose of 50 Gy administered once daily (2 Gy per fraction) (Figure 1d). No serious adverse events, such as perforation of the digestive tract, radiation-induced enterocolitis, or hematuria, were observed. Following the administration of the radiotherapy, the patient underwent one cycle of DXR and IFM chemotherapy followed by single-agent chemotherapy consisting of one cycle of DXR (60 mg/m^2^ on day 1) and one cycle of IFM (2,000 mg/m^2^ on days 1–5) and then was observed. More than 17 months of progression-free survival and sustained symptom relief was achieved.

## Discussion

Leiomyosarcomas are one of the most aggressive subtypes of RPS. PLS was reported to account for 8.6% of all leiomyosarcomas and exhibited an aggressive course accompanied by rapid local recurrence and distant metastasis [3]. A study of 23 PLS patients revealed that 65% died of the tumor and that 48% had recurrence or metastasis. Tumors of the PLS showed morphologically higher grade, and higher mindbomb E3 ubiquitin protein ligase 1 (MIB-1) labeling index than in ordinary leiomyosarcoma patients [3]. In another analysis of 41 cases of leiomyosarcomas with pleomorphic features, metastasis was observed in 89% of cases, and the mortality rate was 50% at the last follow-up (range, 3–104 months; mean, 27.5 months), even though surgical resection and perioperative therapies were performed in 90% and 17%, respectively [4]. The maximum tumor dimension in these studies ranged from 2.5 to 21.0 cm (mean, 9.8; median, 9.0 cm) [4] and from 2.0 to 18 cm (mean, 9.1; median, 9.0 cm) [3]. The histopathological findings of the present PLS case also showed high-grade malignant and proliferating features. However, prominent tumor growth was strikingly observed in which approximately 2 cm of the tumor remaining after debulking surgery enlarged to 30 cm in diameter in only 38 days. These clinical observations strongly suggested an aggressive tumor course and a poor prognosis.

Radiotherapy is a promising modality for the treatment of leiomyosarcoma [5,6]. At the time of initial diagnosis, however, preoperative radiation was not conducted, and the surgical resection was performed for the purpose of quick symptom relief and conservation of organ function. Similar to the favorable results of perioperative radiation for sarcomas of the extremities [7,8], retrospective studies revealed that neoadjuvant radiation therapy for RPS was associated with favorable outcomes [9,10]. While the typical preoperative dose for sarcomas of the extremities is 50 Gy, most institutions use 45–50 Gy of radiation in the treatment of RPS for preoperative radiotherapy. Despite the increased prevalence of multiple risk factors, preoperative radiation did not increase 30-day postoperative morbidity and mortality in a retrospective study of 696 RPS patients [11]. Although a phase III prospective study (Z9031) addressing the role of preoperative radiation for patients with RPS did not report definitive results because of poor accrual [12], another phase III study comparing surgery alone versus perioperative radiation followed by surgery for non-metastatic retroperitoneal sarcoma is now ongoing [13]. These studies could provide us with vital information to help guide appropriate perioperative managements for patients with RPS.

In a retrospective analysis of 500 RPS, 185 patients underwent complete resection and had a significant survival benefit when compared with patients with incomplete resection [1]. Although complete tumor resection could not be achieved in the present case; rather, tumor debulking was performed, adequate complete resection of RPS is the primary goal, and the clinical benefit of debulking surgery has not been proven.

Single-agent DXR is active in the treatment of metastatic STS and is associated with an increased overall response rate (ORR) [14]. While no significant improvements in overall survival (OS) have been reported, combination chemotherapy, such as DXR plus IFM, produces a favorable response and is suggested to be used for the specific condition that tumor response might provide clinical benefit [15]. We employed combination chemotherapy and subsequent radiotherapy (50 Gy/25 fractions) against a reduced-sized tumor, and this strategy resulted in symptom relief and progression free survival for 17 months. Since the efficacy of continuing chemotherapy until disease progression has not been thoroughly investigated [16], considering the cumulative toxicity of DXR and the late toxicity of radiation, close follow-up was chosen in this case. In terms of radiation in combination with chemotherapy, retrospective analysis suggested that ifosfamide pulse radiation had a better outcome than radiation alone [17].

Several new agents for STS, including leiomyosarcoma, are under investigation. The combination of gemcitabine and docetaxel has been shown to possess activity against leiomyosarcoma, particularly uterine lesions (ORR, 58%) [16,18]. Recently, pazopanib, a multi-targeted tyrosine kinase inhibitor, has been reported to be effective in patients with advanced non-adipocytic sarcoma who have a history of prior chemotherapy. That study revealed a significant clinical benefit in progression-free survival in favor of pazopanib when compared with placebo, especially in the subgroup with leiomyosarcoma: the hazard ratio was 0.31 (95% Confidence interval (CI): 0.20-0.40) and the proportion of patients experiencing partial response (PR) and stable disease (SD) was 6% and 67%, respectively [19]. Therefore, these regimens may be a treatment option for cases similar to the present case in the future.

Since leiomyosarcomas tend to be resistant to conventional chemotherapy and because varying results are seen in each individual case [16], a universal approach has not yet been defined. In particular, pathologically high-grade tumors, such as PLS, are associated with a significantly poor survival despite a relative high response rate. The use of a multimodal approach is a promising therapeutic strategy for such cases. In the present case, the use of a multimodal approach was successful, likely based on the following points: quick relief of symptoms by debulking surgery, the dose intensity of the induction chemotherapy was maintained despite the patient’s poor general status, and appropriate radiation therapy was administered after chemotherapy.

## Conclusions

Multimodal therapy with debulking surgery, combination chemotherapy, and radiotherapy can successfully control rapidly progressive retroperitoneal PLS. Maintaining dose intensity of the chemotherapy and radiotherapy might contribute to improved tumor control. Careful selection of therapeutic modalities is required for each patient with PLS.

## Consent

Written informed consent was obtained from the patient for publication of this case report and accompanying images. A copy of the written consent is available for review by the Editor-in-Chief of this journal.

## Abbreviations

STS: Soft tissue sarcoma; RPS: Retroperitoneal sarcoma; PLS: Pleomorphic leiomyosarcoma; DTX: Doxorubicin; IFM: Ifosfamide; PS: Performance status; RECIST: New response evaluation criteria in solid tumours; CTC-AE: Common terminology criteria of adverse events; PR: Partial response; SD: Stable disease; MIB-1: Mindbomb E3 ubiquitin protein ligase 1.

## Competing interests

The authors declare that they have no competing interests.

## Authors’ contributions

KS mainly provided care for the patient and preparation of the manuscript. KT, EK, KU, TM, and KI were in charge of treatments. TM, SM, HK, and EB critically discussed therapeutic plans and preparation of the manuscript. All authors read and approved the final manuscript.
